# Mechanical Stimulation *via* Muscle Activity Is Necessary for the Maturation of Tendon Multiscale Mechanics During Embryonic Development

**DOI:** 10.3389/fcell.2021.725563

**Published:** 2021-09-03

**Authors:** Benjamin E. Peterson, Rebecca A. Rolfe, Allen Kunselman, Paula Murphy, Spencer E. Szczesny

**Affiliations:** ^1^Department of Biomedical Engineering, Pennsylvania State University, University Park, PA, United States; ^2^Department of Zoology, School of Natural Sciences, Trinity College Dublin, The University of Dublin, Dublin, Ireland; ^3^Department of Public Health Science, Division of Biostatistics and Bioinformatics, Pennsylvania State University, Hershey, PA, United States; ^4^Department of Orthopaedics and Rehabilitation, Pennsylvania State University, Hershey, PA, United States

**Keywords:** tendon development, multiscale mechanics, mechanical stimulation, collagen fibril, chick embryo

## Abstract

During embryonic development, tendons transform into a hypocellular tissue with robust tensile load-bearing capabilities. Previous work suggests that this mechanical transformation is due to increases in collagen fibril length and is dependent on mechanical stimulation *via* muscle activity. However, the relationship between changes in the microscale tissue structure and changes in macroscale tendon mechanics is still unclear. Additionally, the specific effect of mechanical stimulation on the multiscale structure-function relationships of developing tendons is also unknown. Therefore, the objective of this study was to measure the changes in tendon mechanics and structure at multiple length scales during embryonic development with and without skeletal muscle paralysis. Tensile testing of tendons from chick embryos was performed to determine the macroscale tensile modulus as well as the magnitude of the fibril strains and interfibrillar sliding with applied tissue strain. Embryos were also treated with either decamethonium bromide or pancuronium bromide to produce rigid or flaccid paralysis. Histology was performed to assess changes in tendon size, spacing between tendon subunits, and collagen fiber diameter. We found that the increase in the macroscale modulus observed with development is accompanied by an increase in the fibril:tissue strain ratio, which is consistent with an increase in collagen fibril length. Additionally, we found that flaccid paralysis reduced the macroscale tendon modulus and the fibril:tissue strain ratio, whereas less pronounced effects that were not statistically significant were observed with rigid paralysis. Finally, skeletal paralysis also reduced the size of collagen fibril bundles (i.e., fibers). Together, these data suggest that more of the applied tissue strain is transmitted to the collagen fibrils at later embryonic ages, which leads to an increase in the tendon macroscale tensile mechanics. Furthermore, our data suggest that mechanical stimulation during development is necessary to induce structural and mechanical changes at multiple physical length scales. This information provides valuable insight into the multiscale structure-function relationships of developing tendons and the importance of mechanical stimulation in producing a robust tensile load-bearing soft tissue.

## Introduction

Tendons are important soft connective tissues that transfer force from muscle to bone. In general, they have strong and tough tensile load-bearing capabilities that are specialized to their anatomical location and physiological function (e.g., energy storage vs. position control; Kastelic et al., 1978; Ker et al., 2000; Thorpe et al., 2012). These unique mechanical properties arise from a complex hierarchical collagenous structure spanning multiple physical length scales over several orders-of-magnitude (Kastelic et al., 1978). While previous studies have provided insight into how this hierarchical structure (and the resulting mechanical properties) is formed (McBride et al., 1988; Birk et al., 1995; Ansorge et al., 2012; Kalson et al., 2015), several important details remain unclear. In particular, tendons undergo rapid changes in macroscale mechanics during late embryonic (in chicks) or neonatal (in mice) development (McBride et al., 1988; Ansorge et al., 2012). Previous work suggests that this mechanical transformation is due to increases in collagen fibril length (Birk et al., 1995) and mediated by mechanical stimulation *via* muscle activity (Pan et al., 2018). However, no study has investigated the multiscale tensile mechanics of tendons during this period of rapid change, which is necessary to fully understand the structure-function relationships of tendon development. Additionally, the biological mechanisms driving tendon maturation (including the role of mechanical stimulation) are unclear (Huang et al., 2015; Havis et al., 2016; Havis and Duprez, 2020; Tan et al., 2020). Understanding the role of mechanical stimulation in driving the rapid changes in tendon development will identify important mechanobiological principles regarding the formation of load-bearing tissues and may advance techniques for tissue engineering and repairing tendon/ligament injuries.

While the timing may vary, the overall structural and mechanical changes observed during tendon development are conserved across species. At embryonic day 10 (E10) in the chick, tendon progenitor cells organize into longitudinal columns *via* cadherin-mediated cell-cell junctions (Richardson et al., 2007). In this process, tenocytes organize themselves such that their cell processes form aligned longitudinal extracellular channels (Kalson et al., 2015). Short (<50 μm) collagen fibrils are then deposited into these extracellular channels *via* fibripositors to form a dense and highly aligned structure (Canty et al., 2004). Up to E16 (HH42), the collagen fibrils exhibit minimal increases in length despite consistent collagen deposition (McBride et al., 1988; Birk et al., 1995). Interestingly, at E17 (HH43) the collagen fibrils undergo an abrupt increase in both their length and diameter, which coincides with a rapid change in tendon macroscale mechanics that continues up to (and beyond) hatching (McBride et al., 1988). The same process is observed postnatally (P0 – P28) in mice (Ansorge et al., 2012; Kalson et al., 2015), and the coincident timing suggests that the changes in collagen fibril structure explain the changes in tendon macroscale mechanics. However, since collagen fibril lengths have not been measured in chick embryos beyond E17 (HH43), the role of this structural parameter on the mechanical properties of developing tendons is still unclear. Additionally, it is unclear how this reorganization of the collagenous network alters the local mechanical environment for tendon cells and how changes in mechanical stimulation drive tendon development.

Existing data suggest that mechanical stimulation *via* muscle activity plays a crucial role in driving the development and maturation of functional load-bearing tendon (Gaut and Duprez, 2016; Havis et al., 2016; Pan et al., 2018; Havis and Duprez, 2020). Mouse embryos or limb bud grafts lacking skeletal muscles show degenerated or absent tendons (Kardon, 1988), and muscle paralysis in chick embryos from E6 to E18 reduces tendon size (Germiller et al., 1998). Interestingly, motility peaks in chick embryos starting at E12 (Wu et al., 2001) and in neonatal rats at P10 (Theodossiou et al., 2019), suggesting that muscle activity may play a particularly important role in driving the rapid structural and mechanical changes observed during late tendon development. Indeed, a recent study demonstrated that immobilizing the developing chick embryo at later stages (HH43; generally equivalent to E17) resulted in a reduction in the compressive modulus of the calcaneal tendon measured *via* atomic force microscopy (Pan et al., 2018). While collagen content was unchanged, muscle immobilization down-regulated the expression of the collagen crosslinker lysyl oxidase (Pan et al., 2018). However, the effect of immobilization on the length and structure of the collagen fibrils is unclear. Furthermore, mechanical testing of tendons from postnatal (P10) rats with reduced muscle activity *via* spinal cord transection showed an *increase* in the tensile modulus (Theodossiou et al., 2021). Therefore, it is still unclear how mechanical stimulation affects the structural maturation of collagen fibrils and the tensile mechanics of tendon during late development.

The objectives of this study were to evaluate the multiscale tensile mechanics of tendon during late embryonic chick development and to identify the effects of skeletal muscle paralysis. Specifically, we simultaneously measured the macroscale tensile mechanics and the deformations of the collagen fibrils (i.e., tensile strain and interfibrillar sliding) in tendons from chick embryos at different developmental stages. We hypothesized that the increase in tendon macroscale mechanical properties observed during development would coincide with an increase in the fibril strains and a reduction in interfibrillar sliding, which is consistent with increasing fibril lengths (Szczesny and Elliott, 2014a). Additionally, we hypothesized that skeletal muscle paralysis would retard the changes in both the fibrillar deformations (i.e., strains and sliding) and the macroscale tensile properties observed during late embryogenesis. Finally, we hypothesized that the changes in tendon hierarchical structure [i.e., cross-sectional area (CSA), size of fibril bundles/fibers] observed during late embryonic development would also be retarded by muscle paralysis. These findings will provide valuable insight into the structural mechanisms that drive tendon development and the role of mechanical stimulation in producing a robust tensile load-bearing tissue.

## Materials and Methods

### Chick Embryo Incubation and Manipulations

Chick embryos were used for this study since their relatively large size and accessibility simplifies mechanical testing procedures and experimental perturbations of muscle activity. Fertilized eggs (White Leghorn or Ross 308) were obtained from their respective suppliers (Poultry Education and Research Center, Pennsylvania State University or Allenwood Broiler Breeders, Kildare, Ireland) and incubated at 37.7°C. All work at the Pennsylvania State University was approved by the Institutional Animal Care and Use Committee. Work on chick embryos does not require a license from the Irish Ministry of Health under European Legislation (Directive 2010/63/EU); all work was approved by the Trinity College Dublin Ethics committee.

**FIGURE 1:**
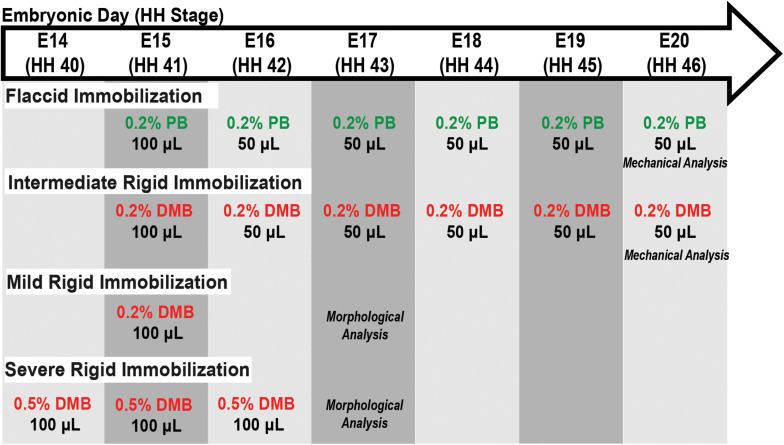
Schematic of immobilization treatment regimens, as indicated by the specific drug and their respective concentration, volumes, and embryonic day (E) in which treatment was administered. Embryos sacrificed for either mechanical or morphological analysis as indicated. Abbreviations: HH, Hamburger and Hamilton stage; DMB, Decamethonium Bromide; and PB, Pancuronium Bromide.

For embryo immobilization, the eggs were windowed at E3 (∼HH20). Briefly, 3–5 ml of albumin was removed from each egg using a wide gauge needle (16$\frac{1}{2}$). Scissors were then used to make a ∼2 cm diameter window in the upper surface of the egg, which was covered with transparent tape to make an airtight seal. Eggs were checked daily to remove any unviable embryos. Immobilization was induced by dripping a treatment solution on the chorioallantoic membrane through the eggshell window. Rigid paralysis of the skeletal muscles was induced by treatment with the neuromuscular blocking agent decamethonium bromide (DMB; Sigma Aldrich; Bowman and Rand, 1980; Mitrovic, 1982; Osborne et al., 2002; Pitsillides, 2006). As described in Figure 1, three treatment regimens were used for rigid paralysis [(mild) 50 μl of 0.2% DMB on E15 (HH41); (intermediate) 100 μl of 0.2% DMB at E15 followed by daily administration of 50 μl 0.2% DMB from E16 (HH42) – E19 (HH45); and (severe) 100 μl of 0.5% DMB from E14 (HH40) – E16 (HH42)]. Flaccid paralysis was induced by treatment with pancuronium bromide (PB; MP Biomedicals; Reiser et al., 1988; Osborne et al., 2002; Pitsillides, 2006) *via* 100 μl 0.2% PB at E15 (HH41) followed by daily doses of 50 μl from E16 (HH42) – E19 (HH45). All solutions were prepared in sterile Hank’s Balanced Salt Solution (HBSS) with 1% antibiotic/antimycotic (penicillin, streptomycin, amphotericin B; Sigma Aldrich). Control embryos were treated with HBSS and antibiotic/antimycotic alone.

Visual inspection of embryonic motility was used to assess musculoskeletal activity during development to determine the effectiveness of the treatments (modified from Wu et al., 2001). Control, rigid paralysis, and flaccid paralysis treated embryos (*n* = 5–6) were randomly selected for motility assessments from E15 (HH41) – E19 (HH45). Briefly, embryos were visually examined in the incubator daily through the transparent window over a 2-min interval in real-time and any discernable individual movement (i.e., kicking or twisting) was counted. Motility counts (events/2 mins) were averaged within each group to get a representative value for each treatment condition per day. Embryos were sacrificed either by sustained exposure to −20°C followed by cervical dislocation or by decapitation and immediate placement in ice-cold phosphate buffered saline (PBS). All embryos were staged using Hamburger and Hamilton criteria (Hamburger and Hamilton, 1951) to ensure proper developmental comparisons as represented in Figure 1.

### Multiscale Mechanical Testing

#### Sample Preparation

Flexor digitorum brevis (FDB) digit II tendons (Supplementary Figure 1) from control embryos sacrificed at E16 (HH42; *n* = 6), E18 (HH44; *n* = 7), and E20 (HH46; *n* = 7) were used to assess the multiscale mechanical changes during late embryonic development. Tendons (*n* = 7) from intermediate DMB and PB immobilized embryos were sacrificed at E20 (HH46), along with vehicle controls, to investigate the effect of immobilization during the same timeframe (Figure 1). Hindlimbs were dissected and the FDB digit II tendon was isolated under a stereomicroscope (Nikon SMZ455T). Upon harvesting, each sample was dragged through wet lens paper with light pressure to remove the paratenon sheath and then stained with dichlorotriazinylaminofluorescein (5-DTAF, Invitrogen) by incubating in a 5 μg/ml solution of 5-DTAF and 0.1 M sodium bicarbonate buffer (pH 9.0) for 10 min at room temperature (Peterson and Szczesny, 2020). After incubation, samples were washed with PBS for 10 min to remove unbound stain.

#### Mechanical Testing

A 10 mm spacer was placed between two custom grips (Peterson and Szczesny, 2020) and a small amount of cyanoacrylate glue (Loctite 454) was placed on each grip face. Each stained sample was carefully lowered onto the grips such that the tarsometatarsal region of the tendon spanned the 10 mm gage length with at least 5 mm of tissue within each grip face. Additional cyanoacrylate was added to each grip face and a drop of adhesive accelerator (Loctite 713) was used to “pot” the tissue ends. Finally, a compression plate was added to each grip and tightened to ∼5 inch-pounds of torque while maintaining tissue hydration with PBS.

The gripped samples were transferred to a custom uniaxial tensile-testing device mounted atop an inverted confocal microscope (Nikon A1R HD) with a PBS bath to ensure adequate hydration during testing. A preload of 0.1 g was applied, and the grip-to-grip distance was measured to establish the initial reference length. Sets of photobleached lines (PBLs; 4 lines, 80 μm apart, 3 μm wide) were bleached at the sample center and ±1.5 mm from the center (Supplementary Figure 2). Reference *z*-stack images were captured (2.5 μm *z*-step, 0.66 μm/px resolution) at each of the three PBL sites. The sample profile in the volumetric images was used to calculate the sample CSA at all three PBL locations assuming an elliptical cross-section, which were averaged to determine a single CSA value. Samples were then stretched in 2% grip-to-grip strain increments at 10%/min followed by a 20-min stress relaxation period. A prolonged relaxation period was applied to ensure that the applied stress values were stable prior to imaging (Peterson and Szczesny, 2020). At the end of each relaxation period, *z*-stack images were acquired at all PBL locations, and the confocal stage positions were recorded. Samples were then incrementally loaded, following the same procedure, until failure.

#### Image Processing and Data Analysis

Custom image processing code (MATLAB, Mathworks) was used to determine the bulk macroscale tissue strains and the microscale fibril strains from the PBL *z*-stack images as previously reported (Peterson and Szczesny, 2020). Briefly, a Sobel edge detection strategy was utilized to create a two-dimensional projection of the curved tendon surface. At every position along the tissue width, the locations of the PBLs were identified as the pixel with the lowest local intensity value. For each strain increment and at each of the three PBL locations (sample center and ±1.5 mm), the microscale fibril strains were measured as the change in distance between the PBLs at every pixel location across the tissue width. Note that these measurements are representative local averages of the actual fibril strains since our imaging resolution was 0.66 μm/px while the average diameter of collagen fibrils in embryonic chick tendons ranges from 45 to 60 nm (McBride et al., 1988). The measured fibril strains were then averaged across all pixels within a given set of PBLs, as well as between all three PBL locations, to generate a single representative value for the fibril strains after each loading increment. To account for potential gripping artifacts, the macroscale bulk tissue strains were optically calculated utilizing the displacement of the peripheral (±1.5 mm) PBL sets. The fibril:tissue strain ratio was calculated at each applied strain increment by dividing the average fibril strain over the bulk tissue strain. Previous work from our lab has demonstrated that a fibril:tissue strain ratio less than one is expected for discontinuous fibrils and that the strain ratio will approach a value of one as the fibril lengths increase (Szczesny and Elliott, 2014a). The level of interfibrillar sliding was quantified by the tortuosity (i.e., waviness) of the PBLs. Briefly, the angle of each PBL (relative to the transverse axis of the tendon) was calculated at each pixel location along the tissue width and the standard deviation of these angles was calculated to characterize the representative amount of interfibrillar sliding (further details provided in Szczesny and Elliott, 2014a). Interfibrillar sliding was calculated after each strain increment at all PBL sites and averaged to obtain a single value.

The equilibrium modulus and ultimate tensile strength (UTS) were calculated to determine the macroscale mechanical properties of the samples. A moving average (window size = 30 data points) was used to smooth the output from the load cell. The equilibrium stress value for each strain increment was calculated by averaging the last 30 s of data within the 20-min stress relaxation period and was plotted vs. the optically tracked bulk tissue strains. The equilibrium modulus was calculated individually for each sample by fitting a line through the second loading increment and the increment preceding the UTS in an effort to minimize the contributions of the toe and failure regions, respectively. Individual sample moduli were then averaged to get a representative modulus for the group. The UTS was defined as the peak equilibrium stress value over the course of the test.

### Morphological Analysis

#### Sectioning and Staining

Embryos were sacrificed daily from E15 (HH41) to E20 (HH46) to assess the structural changes that occur during late tendon development. Control and immobilized embryos (mild and severe treatment regimens; Figure 1) were sacrificed at E17 (HH43) to investigate the effects of immobilization. Briefly, the hindlimbs (distal to the knee joint) from each specimen were processed for paraffin wax sectioning. A full series of longitudinal sections (8 μm) or cross sections (8 μm) through the midpoint of the tarsometatarsal region of interest (ROI) were prepared for each specimen (Supplementary Figure 1). Sections were dewaxed, rehydrated, and stained to highlight connective tissue using Masson Trichrome (Sigma-Aldrich, HT15) or a fluorescently tagged natural pan-collagen binding protein (CNA35-eGFP; Aper et al., 2014). Plasmid DNA encoding enhanced green fluorescent protein (eGFP) fused to the collagen binding protein (CNA35-eGFP) was kindly provided by the laboratory of Maarten Merkx, processed and purified as previously described (Mohammadkhah et al., 2017). Antigen retrieval was performed on histological sections using 0.01 M sodium citrate (pH 8) for 20 min at 90°C prior to blocking of non-specific binding using 1% bovine serum albumin in PBS for 1 h at room temperature. Slides were incubated overnight at 4°C with CNA35-eGFP (1–2 mg/ml) at a dilution of 1:20–1:50 in blocking solution. Post-antibody washes were performed in PBS and counterstained with DAPI. Histological specimens were photographed using an Olympus DP72 camera and CellSens software (v1.6). Confocal microscopy was carried out on a Leica SP8 scanning confocal using a 20X magnification objective. The confocal stack was processed and analyzed using ImageJ (v2.1.0/1.53c) software.

#### Measurement of Morphological Parameters

The cross-sectional areas of the flexor digitorum longus (FDL) and the FDB digit II tendons were measured from embryos across embryonic days E15 (HH41) – E19 (HH45; *n* = 2 for each, except E17 (HH43), *n* = 5) using measurements from 7 to 14 adjacent Masson Trichrome stained cross-sections through a 700–1,300 μm portion of the medial tarsometatarsal ROI (Supplementary Figure 1). The effects of immobilization were assessed at E17 (HH43; *n* = 3 for control, severe, and mild treatment regimens), taking measurements from 5 to 11 adjacent sections per specimen. The boundary of collagen positive (blue) staining at the bundle edge was defined as the boundary of the tendon for quantification. The cross-sectional area of the FDL and FDB tendons were quantified independently using ImageJ (v2.1.0/1.53c).

The same medial ROI was used to assess the spacing between the FDL and FDB tendons over time and following immobilization (Supplementary Figure 1). For each section from each sample, 6–10 measurements were taken across the extent of the interface (where approximately parallel) between the tendons, and averaged, to represent the spacing between the tendons (Supplementary Figure 1C; red lines indicate typical regions for measurement).

The collagen fiber diameters were estimated from confocal fluorescence volumetric images of the CNA35-eGFP stain across developmental stages and following immobilization. Two to three image planes from 5 to 9 ROIs (100 μm^2^) per specimen were analyzed, which provided a measurement of 79–117 individual fibers per specimen [immobilization experiment (E17, HH43): *n* = 6 control, *n* = 4 severely immobilized, and *n* = 2 mildly immobilized] (Figure 1). Individual fibers in the 100 μm^2^ ROI were measured using ImageJ software from edge to edge (Supplementary Figure 1) as determined by a higher intensity of CNA35-eGFP fluorescence. To assess fiber alignment, the ImageJ FibrilTool plugin (Boudaoud et al., 2014) was used to measure the level of anisotropy on 9–11 regions of interest per confocal image (100 μm^2^), averaged for each biological sample (control *n* = 5; mild immobilization *n* = 2; and severe immobilization *n* = 4). An anisotropy score of 0 indicates a fully random/isotropic fiber organization and a score of 1 indicates a perfectly ordered organization, i.e., parallel fibers.

### Statistical Analysis

Significance was set at *p* ≤ 0.05 for all tests. For mechanical characterization, statistical analysis was conducted using SAS (9.4) and GraphPad Prism (8.3.0). A one-way ANOVA was conducted to evaluate the UTS and equilibrium modulus with respect to development or paralysis treatment. *Post hoc* Tukey’s tests corrected for multiple comparisons were conducted to evaluate the UTS and modulus with increased maturation. A *post hoc* Dunnett’s test corrected for multiple comparisons evaluated the effect of immobilization treatments compared to the vehicle control. An ANCOVA with Bonferroni *post hoc* tests was used to determine if the embryonic motility was affected by development or immobilization treatment. Differences in the fibril:tissue strain ratio and interfibrillar sliding were evaluated using a linear mixed model that takes into consideration the correlated strain increments per specimen as a covariate (Diggle et al., 2002). A linear regression was conducted to test whether the fibril:tissue strain ratio was negatively correlated with applied tissue strain, and a one-sample *t*-test was conducted to determine if the group mean was significantly less than 1. Similarly, a linear regression was conducted to test whether the fibril sliding behavior was positively correlated with applied tissue strain. For morphological analysis, differences in tendon area, distances between tendons, and fiber diameter were assessed by univariate ANOVA followed by Tukey *post hoc* tests using SPSS (SPSS Statistics v26, IBM). Fiber bundle anisotropy was evaluated by a one-way ANOVA with a Dunnet’s *post hoc* test corrected for multiple comparisons.

## Results

### Changes in Tendon Multiscale Mechanics and Structure With Development

**FIGURE 2:**
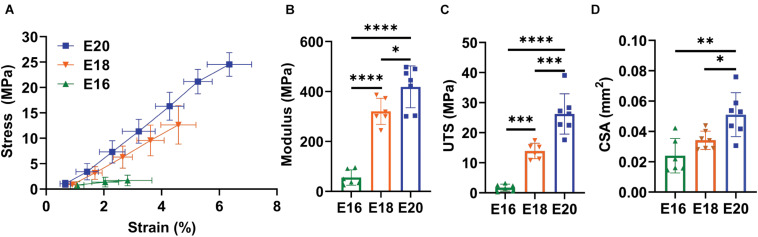
Flexor digitorum brevis (FDB) digit II tendon macroscale mechanical response with maturation. **(A)** Equilibrium stress vs. strain response for E16 (HH42, *n* = 6), E18 (HH43, *n* = 7), and E20 (HH46, *n* = 7) timepoints. **(B)** Significant increase in equilibrium modulus response with embryonic maturation. **(C)** Significant increase in ultimate tensile stress (UTS) with development. **(D)** A significant increase in the cross sectional (CSA) of the tendon was observed with maturation when measured under confocal microscopy. Significance was tested with a one-way ANOVA with *post hoc* Tukey’s test corrected for multiple comparisons. Data represented as mean ± standard deviation. ^∗^*p* < 0.05, ^∗∗^*p* < 0.01, ^∗∗∗^*p* < 0.001, and ^*⁣*⁣**^p < 0.0001.

There was a significant increase in the macroscale modulus with development (Figure 2B; *p* < 0.0001) with significant differences observed between E16 (HH42) and both E18 (HH44; *p* < 0.0001) and E20 (HH46; *p* < 0.0001) as well as between the E18 (HH44) and E20 (HH46) timepoints (*p* < 0.05). The UTS was also significantly increased at later developmental timepoints (Figure 2C; *p* < 0.0001) with similar individual differences between the E16 (HH42) and both E18 (HH44; *p* < 0.001) and E20 (HH46) timepoints (*p* < 0.0001) as well as between the E18 (HH44) and E20 (HH46) timepoints (*p* < 0.001). Finally, there was a significant increase in the tendon cross-sectional area as measured by confocal microscopy with development (Figure 2D; *p* < 0.01). *Post hoc* comparisons found a significant difference between E16 (HH42) and E20 (HH46; *p* < 0.01) as well as between E18 (HH44) and E20 (HH46; *p* < 0.05) but no difference the E16 (HH42) and E18 (HH44) samples. These large changes in macroscale mechanics are consistent with prior data showing increases in mechanical properties during late tendon development (McBride et al., 1988).

**FIGURE 3:**
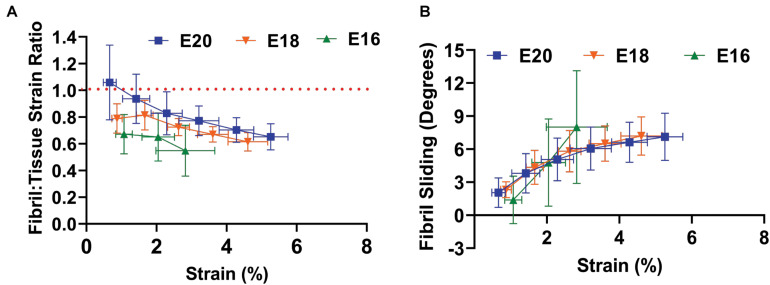
Multiscale mechanical response of the flexor digitorum brevis (FDB) digit II tendon as a function of applied tissue strain with increasing development. **(A)** A significant increase in the fibril: tissue strain ratio was observed between E16 (HH42) vs. E18 (HH43; *p* < 0.05) and E16 (HH42) vs. E20 (HH46; *p* < 0.001). **(B)** No significant difference in the fibril sliding behavior was observed with developmental age. Significance was tested with a linear mixed model accounting for correlated strain increments per specimen as a covariate. Data represented as mean ± standard deviation.

To assess the mechanisms underlying these macroscale mechanical changes during development, we measured the fibril:tissue strain ratio and interfibrillar sliding as a function of applied tissue strain. Previous work has demonstrated that a fibril:tissue strain ratio less than one is expected for discontinuous fibrils and that the fibril:tissue strain ratio will increase (while the interfibrillar sliding will decrease) as the collagen fibrils elongate (Szczesny and Elliott, 2014b). All embryonic samples, regardless of their developmental timepoint, displayed a mean fibril:tissue strain ratio that was less than one and negatively correlated with the applied tissue strain (Figure 3A; Control: *p* < 0.05, PB: *p* < 0.0001, and DMB, *p* < 0.001). A linear mixed model found a significant increase in the fibril:tissue strain ratio between E16 and both the E18 (HH44; *p* < 0.05) and E20 (HH46; *p* < 0.001) timepoints. Additionally, the interfibrillar sliding was positively correlated with the applied tissue strains for all samples (Figure 3B). However, there were no significant difference in the interfibrillar sliding with development.

**FIGURE 4:**
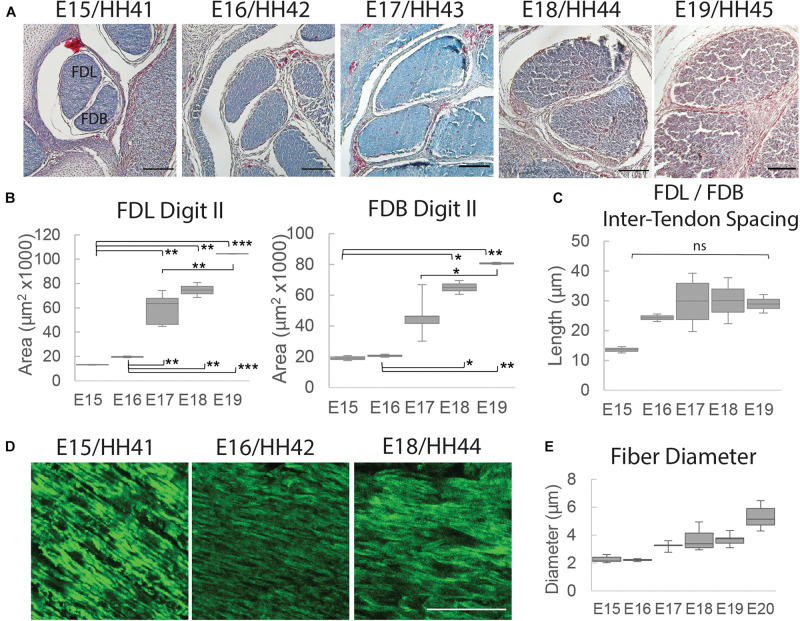
Structural analysis of tendons between E15 (HH41) and E19 (HH45). **(A)** Cross-sectional profile of the flexor digitorum longus (FDL) and brevis (FDB) digit II tendons stained with Masson Trichrome in the tarsometatarsal region of the developing chick hindlimb over embryonic days, as indicated. Scale bar 100 μm. **(B)** Box plots of cross-sectional area of the FDL and FDB digit II tendon over developmental time (plot whiskers indicate min and max data values). **(C)** Box plot of spacing measurements between the FDL and FDB digit II tendons over developmental time (measurements taken through a 700 – 1,330 μm portion of the medial tarsometatarsal region, see Supplementary Figure 1). **(D)** Images of longitudinal tendon fibers in the tarsometatarsal region stained with collagen binding protein over developmental time. Scale bar 50 μm. **(E)** Box plot of tendon fiber diameter measurements over time. Statistical analysis was carried by univariate ANOVA followed by Tukey *post hoc* tests. For **(B)** *n* = 2 per day except E17 (HH43) *n* = 5; For **(E)** *n* = 1 (multiple sections from 1 specimen/stage measured). *p ≤ 0.05, **p ≤ 0.01, and ***p ≤ 0.001.

To profile the morphological changes in the FDL and FDB tendons over the developmental time window, transverse histological sections through the tendon midpoint were analyzed (Supplementary Figure 1 and Figure 4A). An increase in tendon size was observed through measurements of the cross-sectional area (Figure 4B); in both tendons there was a steady increase in the mean value during development with a larger step increase at E17 (HH43), particularly for the FDL. The distance (spacing) between the tendons did not change significantly over time, but there was a visible increase between E15 (HH41) and E16 (HH46; Figure 4A), reflected in the mean distance measurement (Figure 4C). Longitudinal sections, stained with the fluorescently tagged pan-collagen binding protein CNA35-eGFP, allowed visualization of the underlying collagen network across time (Figure 4D). The fibril bundles (fibers) visibly enlarged, reflected in an incremental increase in the average fibril bundle diameter over time (Figure 4E). There was no obvious change in the alignment of the fibril bundles over time, at least at this level of resolution.

### Effect of Muscle Paralysis on Tendon Development

**FIGURE 5:**
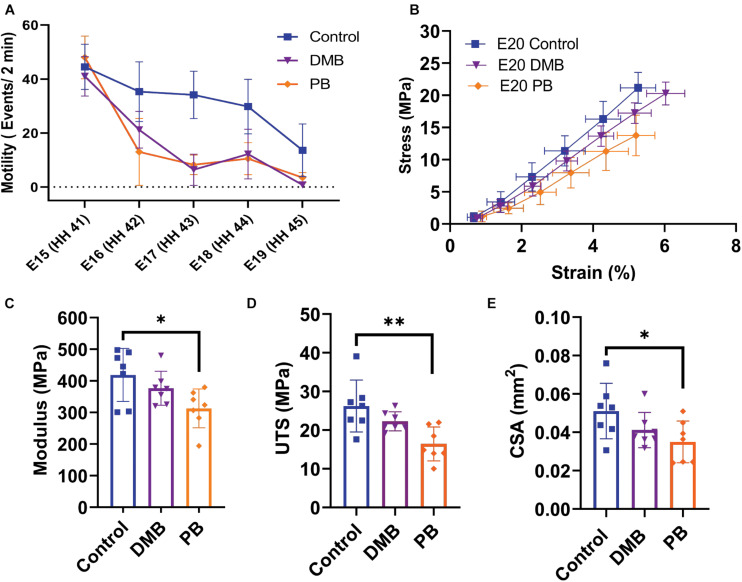
Muscle paralysis and its effect on the macroscale mechanical properties of the flexor digitorum brevis (FDB) digit II tendon during development. **(A)** Paralysis treatments and saline control were conducted from E15 (HH41) – E19 (HH45). Significant decrease in motility observed under rigid (DMB, *p* < 0.05) and flaccid (PB, *p* < 0.05) paralysis. No significant difference in motility counts between the two paralysis methods. **(B)** Equilibrium stress vs. applied strain for E20 (HH46) samples after control (*n* = 7), rigid paralysis (DMB, *n* = 7), and flaccid paralysis (PB, *n* = 7) treatments. **(C)** Equilibrium modulus is significantly reduced with flaccid paralysis treatment (*p* < 0.05). **(D)** Significant decrease in ultimate tensile stress (UTS) with flaccid paralysis (*p* < 0.01). **(E)** Cross-sectional area of flexor digitorum brevis (FDB) tendon was observed under flaccid paralysis conditions when measured under confocal microscope (*p* < 0.05). Effects on embryonic motility were evaluated using an ANCOVA with Bonferroni *post hoc* test. Significance for Modulus, UTS, and CSA were evaluated using a one-way ANOVA with *post hoc* Tukey’s test corrected for multiple comparison. Data represented as mean ± standard deviation ^∗^*p* ≤ 0.05, ^∗∗^*p* ≤ 0.01.

Rigid and flaccid paralysis treatment, with DMB (intermediate regimen) and PB, respectively, significantly reduced embryonic motility through the target period (DMB: *p* < 0.05, PB *p* < 0.05; Figure 5A). No significant difference in motility was observed between the rigid DMB or flaccid PB paralysis treatments (*p* = 0.67). After validating the immobilization treatments, we investigated their effect on the mechanical and structural changes that occur during late embryonic development (Figures 2, 3). At the macroscale level, flaccid paralysis (PB treatment) significantly reduced the equilibrium modulus compared to vehicle control samples (Figure 5C; *p* < 0.05). Similarly, flaccid paralysis significantly decreased both the UTS (*p* < 0.01) and CSA (*p* < 0.05; Figures 5D,E). No significant differences in the macroscale mechanical properties were observed under the rigid paralysis (DMB) conditions compared to controls (modulus: *p* = 0.47, UTS: *p* = 0.24, and CSA: *p* = 0.22).

**FIGURE 6:**
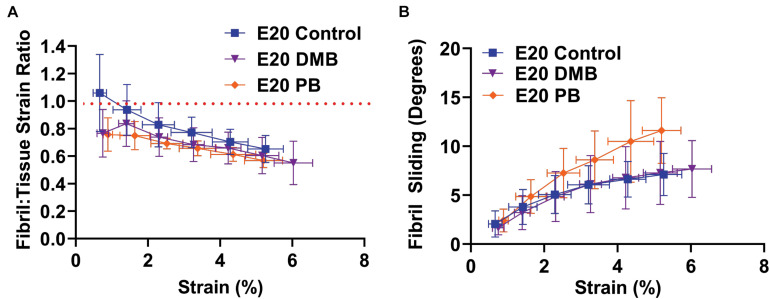
Multiscale mechanical response at E20 (HH46) as a function of applied tissue strain after immobilization treatments. **(A)** The fibril:tissue strain ratio was significantly lower with flaccid paralysis (PB) treatments in comparison to the control tissue (*p* < 0.05). No significant difference was observed between the rigid paralysis (DMB) and the vehicle control tissue (*p* = 0.106). **(B)** All samples displayed an increase in fibril sliding angle with applied stress. No significant group effects were observed under paralysis conditions in comparison to vehicle controls (PB vs. Control: *p* = 0.10, DMB vs. Control: *p* = 0.78). Significance was tested with a linear mixed model accounting for correlated strain increments per specimen as a covariate. Data represented as mean ± standard deviation. Data represented as mean ± standard deviation.

To investigate how the multiscale mechanics were perturbed under paralysis conditions, we measured the fibril:tissue strain ratio and interfibrillar sliding as a function of applied tissue strain for each treatment group. All samples (control, PB, and DMB) displayed a mean fibril:tissue strain ratio that was less than one (*p* < 0.05) and was negatively correlated with applied tissue strain (Figure 6A; *p* < 0.001). A linear mixed model found a significant decrease in the fibril:tissue strain ratio for the flaccid (PB) immobilized samples compared to control tissues (*p* < 0.05). No significant differences were found between the rigid immobilized and control tissue (*p* = 0.11). The interfibrillar sliding was positively correlated with the applied tissue strain for all samples (Figure 6B). However, no significant group effects were observed under paralysis conditions in comparison to the control (PB: *p* = 0.10, DMB: *p* = 0.78).

**FIGURE 7:**
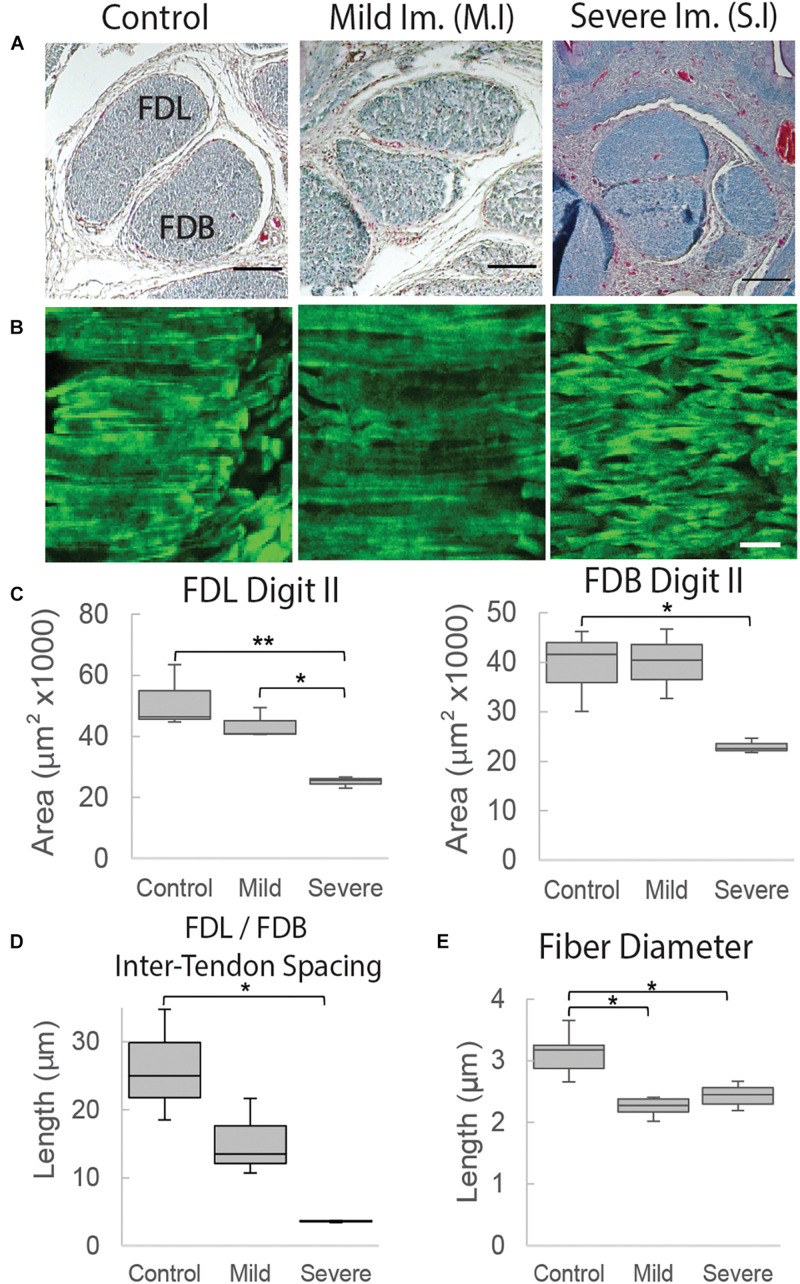
Structural organization of tendons at E17 (HH43) is altered following immobilization by rigid paralysis (DMB treatment). **(A)** Representative cross-sections through the flexor digitorum longus (FDL) and brevis (FDB) digit II tendons stained with Masson Trichrome. Images, from the left, show control treatment, mild Immobilization (Mild Im.) and severe immobilization (Severe Im.) treatment conditions (see Figure 1). Scale bar 100 μm. **(B)** Representative longitudinal sections stained with collagen binding protein (CNA35-eGFP) under the same conditions as **(A)**, as indicated. Scale bar 10 μm. **(C)** Box plots represent cross sectional area measurements of the FDL and FDB digit II tendons showing significant reduction following severe rigid paralysis (plot whiskers represent min and max data values). **(D)** Box plot representing the spacing measurement between tendons with significant reduction under severe immobilization conditions. **(E)** Box plot representing fiber diameter measurements with significant reduction following both mild and severe immobilization treatments. Statistical analysis was carried by univariate ANOVA followed by Tukey *post hoc* tests. *n* = 3, all groups in **(C,D)**; in **(E)**, *n* = 6 (control), *n* = 4 (S.I), and *n* = 2 (M.I). *p ≤ 0.05, **p ≤ 0.001.

Rigid paralysis of chick embryos caused multiple changes to the morphology of the FDL and FDB tendons at E17 (Figure 7). Two regimens of DMB treatment were tested for effect; a more severe treatment that involved 0.5% applied daily from E14 and a milder regimen consisting of a single treatment with 0.2% at E15 (Figure 1). At a gross morphological level, both the cross-sectional area and the spacing between tendons were significantly reduced with the more severe exposure alone (*p* < 0.01). However, a greater sensitivity to treatment was observed at a smaller length scale, with fiber diameter significantly reduced by both mild and severe treatments. While the collagen fibers appeared more disorganized with severe immobilization (Figure 7B), analysis of the fiber orientation anisotropy did not find a statistically significant difference with treatment (Supplementary Figure 3).

## Discussion

This study demonstrated that there is a pivotal shift within tendon structure-function relationships during late stages of embryonic development that facilitates the functional load-bearing capabilities of tendons. Specifically, there is an increase in the fibril:tissue strain ratio after E16 (HH42) in chick embryos (Figure 3A), which is consistent with an increase in the length of the collagen fibrils (Birk et al., 1995; Szczesny and Elliott, 2014b) and the increase in macroscale tensile mechanics (Figure 2; McBride et al., 1988). Similar maturation at the microscale level has also been observed *via* measurement of the compressive mechanical properties of developing chick calcaneal tendons *via* atomic force microscopy (Marturano et al., 2013). It is also important to be note that the E20 (HH46) tissue still displayed a negatively correlated fibril:tissue strain ratio at larger applied strains, which is consistent with discontinuous fibrils and not progressive breakage of continuous fibrils (Peterson and Szczesny, 2020). However, the observed upward shift in the fibril:tissue strain ratio with maturation indicates a transition in the collagenous loading behavior, in which strains are being transferred to the collagen fibrils more directly. While shorter fibrils should exhibit more relative sliding with tissue stretch (Szczesny and Elliott, 2014a,b), no significant difference in interfibrillar sliding was observed between E16 and the later developmental timepoints. We have previously observed similar discrepancies between the change in the fibril:tissue strain ratio (or lack thereof) and the measurement of interfibrillar sliding, which we believe is due to heterogenous tissue loading possibly caused by gripping artifacts (Szczesny et al., 2017). Additionally, the rapid changes in tissue ultrastructure observed at E16 (Birk et al., 1995) is likely responsible for the large variance in the interfibrillar sliding for this developmental timepoint, which may have masked a possible difference in the interfibrillar sliding. Rapid development at this timepoint is further supported by morphological analysis of the FDB and FDL tendons, which showed a significant increase in the CSA (Figure 4B) and an incremental increase in the average fiber diameter with development in the samples assayed (Figure 4E). Together, this study supports our hypothesis that there is a reorganization of the collagenous network during late stages of tenogenesis that increases the strain transmitted to the collagen fibrils and generates a rapid increase in tendon mechanical properties.

Our findings also demonstrate that the observed changes in fibril loading and macroscale tensile mechanics are impeded by musculoskeletal paralysis, suggesting that mechanical stimulation during late stages of tenogenesis is critical to mediating developmental changes in the collagen fibril network. Specifically, flaccid muscle paralysis reduced the macroscale modulus, UTS, and CSA (Figure 5) as well as the fibril:tissue strain ratio (Figure 6) with a trending increase in the interfibrillar sliding (*p* = 0.1). Interestingly, the effect of rigid paralysis on the macroscale mechanics (Figures 5B–E) and fibril loading (Figure 6A) was not significant, despite a comparable decrease in embryo movement as flaccid paralysis treatment (Figure 5A). This variation in response is likely attributed to the different modes of action in which these drugs induce immobilization. PB competitively blocks the acetylcholine receptor at the motor endplate to inhibit muscle contraction, causing flaccid or limp paralysis (Bowman and Rand, 1980; Osborne et al., 2002). In contrast, DMB irreversibly binds to acetylcholine receptors in the motor endplate to induce permanent contraction of muscle, generating a prolonged static load across the tissue (Bowman and Rand, 1980; Osborne et al., 2002). We hypothesize that while both immobilization models reduce cyclical musculoskeletal stimulation, the static loading environment induced by the DMB treatment reduces the overall phenotypic effect. Differences between the effects of rigid and flaccid paralysis on skeletal development have also been noted. Conversely, the effects of rigid paralysis on the spine were more pronounced (Rolfe et al., 2017), as was the effect on length and breadth of the cartilaginous skeletal rudiments, while flaccid paralysis caused greater disruption of post-cavitation stage joints (Osborne et al., 2002). Although the static loading experienced by tissues under rigid paralysis (Nowlan et al., 2008) can have a more detrimental effect under some circumstances, it is clearly not the case for these stages of tendon development. Despite this, rigid paralysis treatments still have a significant effect on the tissue morphology in a dose dependent manner (Figure 7). Histological characterization demonstrated that severe rigid immobilization was required to induce a significant reduction in the CSA and inter-tendon spacing, with minimal effects under a mild treatment paradigm. Interestingly, both the mild and severe rigid immobilization treatments induced comparable reductions in the collagen fiber diameter. Although we did not observe a statistically significant difference in fiber alignment with immobilization, future experiments utilizing polarized light microscopy or Raman spectroscopy are necessary to investigate possible alignment changes more thoroughly. Altogether, our data suggest that ultrastructural changes in the collagen network are sensitive to the loss of external mechanical stimulation, which is consistent with our mechanical data using a similarly mild treatment (Figures 5, 6).

While our data suggest that mechanical stimulation plays an important role in driving the structural and mechanical changes observed during late tendon development, the biological mechanisms mediating this are unclear. Prior work has shown that the loss of mechanical stimulation during late stages of tendon development reduces LOX expression, inhibiting cross-linking activity within the collagenous network and reducing the tissue compressive modulus (Makris et al., 2014; Pan et al., 2018). Furthermore, a recent study has identified that increases in collagenous crosslinking and tissue stiffness is directly regulated by Piezo1 activation *via* shear deformation resulting from fiber/fibril sliding (Passini et al., 2021). This suggests that the effects of mechanical stimulation on tendon development may be mediated by increased collagen crosslinks driven by activation of mechanosensitive ion channels. Still, numerous other ECM components are known to regulate collagen fibril interconnections, and it is not clear how they are affected by mechanical stimulation. For example, decorin, and biglycan bind to the surface of collagen fibrils and inhibit collagen fibril fusion (Schönherr et al., 1995; Graham et al., 2000; Zhang et al., 2006), which may explain the rapid change in collagen fibril structure during late tendon development (Robinson et al., 2005; Zhang et al., 2006). Furthermore, their relative expression decreases during development precisely when fibril lengths/diameters grow rapidly (Birk et al., 1995; Chen and Birk, 2013). However, previous work indicates that decorin expression is positively correlated to mechanical stimulation (Hayashi et al., 2019). This suggests that the reduction in the fibril:tissue strain ratio (potentially due to a retardation in collagen fibril elongation) seen with flaccid paralysis (Figure 7A) may not be due to a change in decorin or biglycan expression. Another important note is that there were still significant increases in tendon macroscale mechanics with PB treatment when compared to normally developing E16 tissue, suggesting that some load-bearing elements may develop independently of mechanosensitive mechanisms. Further investigations are required to characterize the changes in tendon structure-function relationships during late tendon development, its sensitivity to musculoskeletal stimulation, and other regulatory mechanisms that link these two. Overall, this study supports our hypothesis that mechanical stimulation during late tendon development plays a critical role in mediating the gross structural and mechanical changes across multiple length scales.

A limitation to this study is that it took 48 h to produce the full immobilization effects of the paralytic drug treatments. Therefore, it is possible that some mechanosensitive maturation occurred during E15 (HH41) – E17 (HH43) even with treatment, which could explain the increase in mechanical properties of the E20 (HH46) PB treated samples compared to normally developing E16 (HH42) tissue. An additional limitation is that confocal microscopy does not have the optical resolution required to directly visualize collagen fibrils. That is, our imaging resolution is 0.66 μm/px, while the average diameter of collagen fibrils during these developmental timepoints range from 45 to 60 nm (McBride et al., 1988). However, fibril bundles (i.e., fibers) during this same developmental window are approximately 2–5 μm in diameter (Silver et al., 2003), suggesting that we are capturing a localized average fibril response. Finally, changes in fibril structure (e.g., fibril length, diameter, and interfibrillar spacing) and their dependence on musculoskeletal activity during late tendon development were not directly measured. Nevertheless, this study provides valuable insight into the multiscale structure-function relationships of developing tendons and the importance of mechanical stimulation in producing a robust tensile load-bearing soft tissue. While there is limited knowledge translating our findings within chick embryos to humans, we believe that this study provides foundational knowledge into the structure-function mechanisms that dictate critical periods of tendon development.

## Data Availability Statement

The raw data supporting the conclusions of this article will be made available by the authors, without undue reservation.

## Ethics Statement

The animal study was reviewed and approved by Pennsylvania State University Institutional Animal Care and Use Committee and the Trinity College Dublin Ethics Committee.

## Author Contributions

BP and RR carried out the reported experiments and data analysis. AK aided in the design of the statistical analysis. SS and PM supervised the work and contributed to the interpretations of the results and writing of the manuscript. BP was the primary author of the manuscript. All authors provided crucial feedback to shape the research, analysis, and manuscript.

## Conflict of Interest

The authors declare that the research was conducted in the absence of any commercial or financial relationships that could be construed as a potential conflict of interest.

## Publisher’s Note

All claims expressed in this article are solely those of the authors and do not necessarily represent those of their affiliated organizations, or those of the publisher, the editors and the reviewers. Any product that may be evaluated in this article, or claim that may be made by its manufacturer, is not guaranteed or endorsed by the publisher.
