# Supplementary material for: Mechanical Stimulation via Muscle Activity Is Necessary for the Maturation of Tendon Multiscale Mechanics During Embryonic Development
Source: Front Cell Dev Biol. 2021 Sep 3;9:725563. doi: 10.3389/fcell.2021.725563 (PMC8446456; doi:10.3389/fcell.2021.725563)
Supplement: Supplementary file 1 [file Data_Sheet_1.docx]

Supplementary Material

## Supplementary Figures


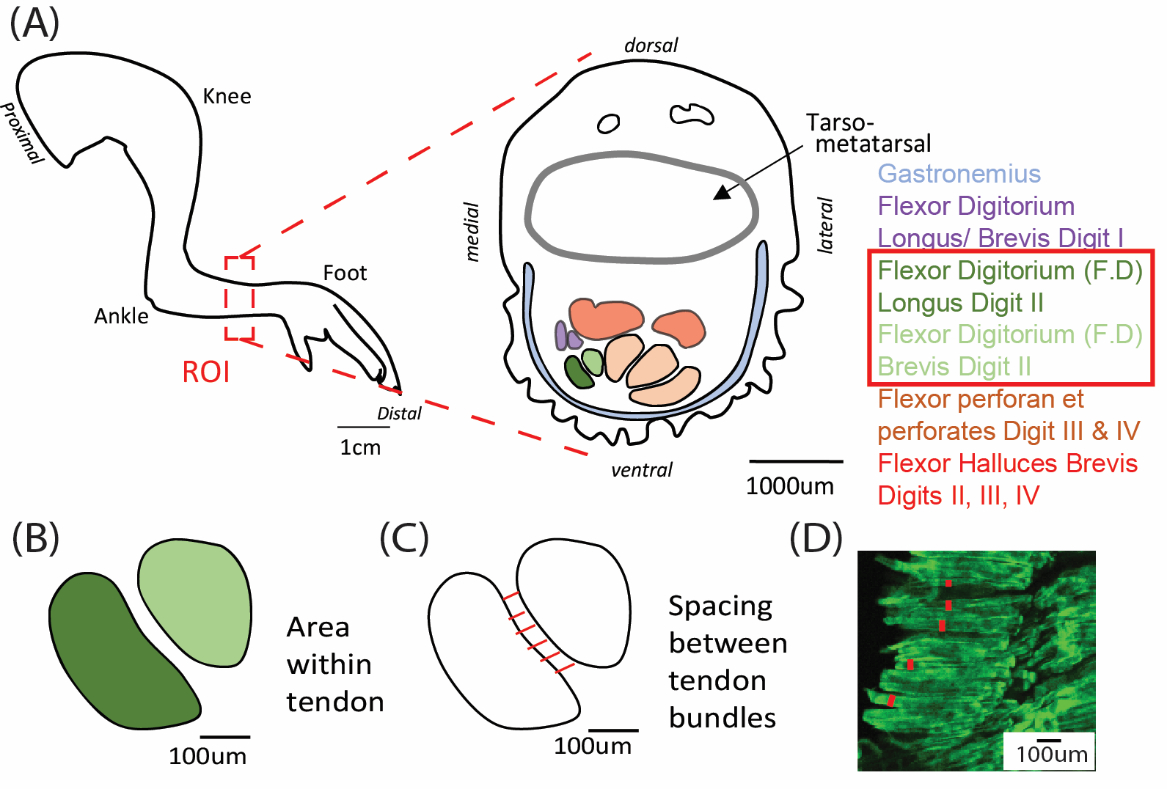


**Supplementary Figure 1.** (A) Schematic of a chick embryonic hindlimb showing a region of interest (ROI) through the tarsometatarsal region, and detailed morphology of cross-sections through this ROI with individual tendons labelled and color matched. (B) Schematic of the flexor digitorium longus and brevis digit II tendons to quantify the cross-sectional area within a tendon, (C) and the spacing between tendons, shown by red lines. (D) Confocal data of collagen binding protein (CNA35-eGFP) stained fibers were used to measure fiber diameter, indicated with individual red lines. All scale bars indicated.


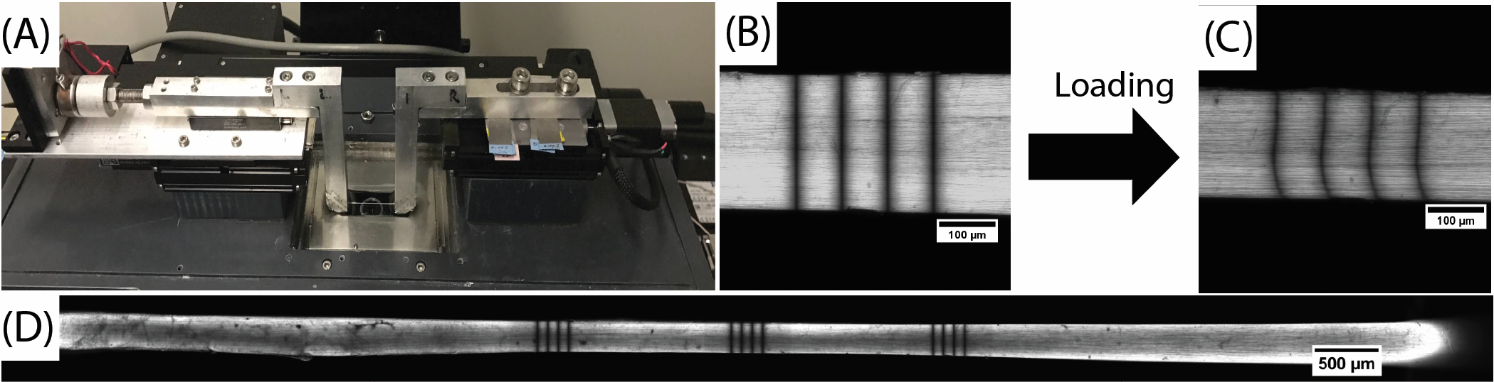


**Supplementary Figure 2.** Experimental setup for multiscale mechanical testing. (A) Custom uniaxial tensile testing device mounted atop a confocal microscope. (B & C) Representative images of a photobleached line (PBL) site at prior to and after loading, respectively. (D) Tendon sample held at a 10 mm gauge length with PBL sets at the sample center and ± 1.5 mm.

**
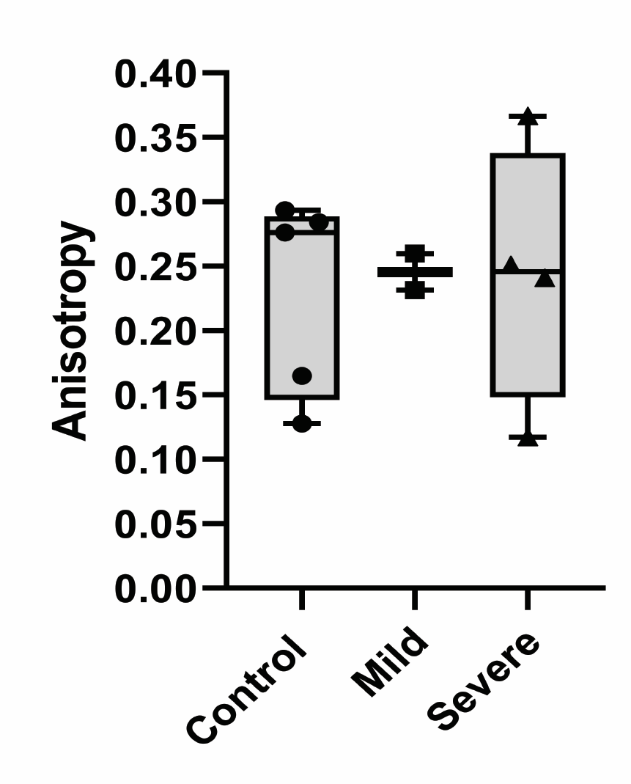
**

**Supplemental Figure 3.** Collagen fiber anisotropy at E17 (HH43) is unaffected following immobilization by rigid paralysis (DMB treatment). Box plot represents fiber anisotropy under mild and severe rigid immobilization (whiskers indicate min/max values). Fiber anisotropy evaluated using a one-way ANOVA with Dunnett’s post-hoc test corrected for multiple comparisons.
